# Rituximab added to conditioning regimen significantly improves erythroid engraftment in major incompatible ABO-group hematopoietic stem cell transplantation

**DOI:** 10.1038/s41409-024-02247-w

**Published:** 2024-02-24

**Authors:** Maria Chiara Finazzi, Alessandra Weber, Chiara Pavoni, Anna Grassi, Maria Caterina Micò, Alessandra Algarotti, Federico Lussana, Alessandro Rambaldi

**Affiliations:** 1 Department of Oncology and Onco-Hematology, Università degli Studi di Milano, Milano, Italy; 2 Hematology and Bone Marrow Transplant Unit, ASST Papa Giovanni XXIII, Bergamo, Italy

**Keywords:** Cancer therapy, Haematological cancer

## Abstract

ABO-group major incompatibility hematopoietic stem cell transplantation (HSCT) increases the risk of delayed red cell engraftment and other immunological complications. In this study, we evaluated the efficacy of pre-transplant infusion of rituximab in patients with ABO-incompatibility in improving red blood cell engraftment after HSCT, measured by time to reach transfusion independence. We performed a retrospective, single-center study including 131 consecutive patients transplanted with major or bidirectional ABO-incompatible grafts between 1st January 2013 and 31st December 2019. Fifty-one patients received an infusion of rituximab during the conditioning regimen, while 80 patients did not receive any additional preventive treatment. Time to transfusion independence was significantly reduced for patients treated with rituximab (1 month, 95% CI, 0.5–2) compared with the control group (3.2 months, 95% CI 1.5–3.2, *p* = 0.02). By multivariable analysis, rituximab use was associated with a faster red blood cell (RBC) engraftment (RR 1.88, 95% CI 1.17–3.03, *p* = *0.009*), while a pre-transplant anti-donor isohemagglutinins titer >1:128 was associated with delayed transfusion independence (RR 0.61, 95% CI 0.37-0.99, *p* = *0.05*). Although limited by the retrospective nature of the study, the results of this analysis suggest that rituximab added to conditioning regimens is feasible, safe, and able to improve post-transplant red blood cell engraftment.

## Introduction

Approximately 40–50% of all allogeneic hematopoietic stem cell transplantation (HSCT) are performed with some degree of ABO blood group incompatibility between the donor and the recipient [1] Three types of ABO mismatches have been described: major, minor, and bidirectional [2]. Although the transplantation of an ABO-mismatched graft is feasible, this condition is at risk for delayed hemolysis, pure red cell aplasia (PRCA) [3] delayed red blood cell (RBC) engraftment [4, 5].

Globally, the clinical outcome of ABO-incompatible HSCT is generally considered inferior to ABO-compatible HSCT. ABO incompatibility may have an impact on platelet and neutrophil engraftment. Since ABO blood group antigens are also expressed on platelets, previous studies reported a significant delay in platelet engraftment [6–8]. Even if AB antigens are not directly expressed by neutrophils, delayed engraftment of neutrophils was also previously reported by other groups [6, 9] speculating that anti-donor isohemagglutinin may bind to A/B antigens adsorbed on the surface of neutrophils or their precursors. ABO incompatibility has been associated with graft failure [6, 10], even if others did not confirm this data [11]. Furthermore, since AB antigens are also expressed in non-hematopoietic tissues, ABO major incompatibility may impact GVHD incidence. Some studies reported an increased incidence of acute graft versus host disease (GVHD) [5, 12–14], even if results are conflicting [5]. Lastly, ABO incompatibility may have a detrimental effect on non-relapse mortality and overall survival [5, 15], especially during the first 100 days after transplant [16].

The prevention of immune complications after an ABO-incompatible transplant relies mainly on the possibility of reducing the isohemagglutinins titer in the recipient before transplant by apheresis; [17] however, this approach is not standardized and there is no consensus about it [2].

Rituximab is an anti-CD20 chimeric IgG1 monoclonal antibody. It has been used in the treatment of post-transplant PRCA to reduce isohemagglutinin titers in the host with some success [18]. Given its effect in reducing the antibody production by B lymphocytes, we hypothesized that pre-transplant infusion of rituximab in patients with ABO-incompatibility between the host and the donor may improve the RBC engraftment.

In this study, we retrospectively analyzed the outcome of 131 patients transplanted in our center with major or bidirectional ABO-incompatible grafts between 1st January 2013 and 31st December 2019. Of these, 51 patients received rituximab during the conditioning regimen, while 80 did not receive additional treatment. We compared these two cohorts of patients to detect differences in time to reach RBC transfusion independence.

## METHODS

### Patients

We performed a single-center retrospective analysis on 131 consecutive patients who received major or bidirectional ABO-incompatible allogeneic HSCT at the Hematology and Bone Transplant Unit, ASST Papa Giovanni XXIII, Bergamo between 1st January 2013 and 31st December 2019. All patients included in this study signed an informed consent to allow the scientific use of their transplant data to the EBMT (European Bone Marrow Transplantation) database [IRB00003888].

### ABO incompatibility prophylaxis

From September 2016 to December 2019, patients with major ABO incompatibility were evaluated for rituximab infusion before transplant. Of the 66 patients transplanted with ABO mismatch after this date, 51 patients received rituximab during the conditioning regimen, while 15 patients did not receive any additional treatment. The decision to omit rituximab in these 15 patients was due to a low anti-donor isohemagglutinins titer before transplant (<1:64). These patients were included in the control group. Similarly, the decision to administer rituximab to 51 patients was made on a combination of factors, including the presence of a high anti-donor isohemagglutinins titer (median titer 1:128) and clinical factors such as the combination of donor-patient blood groups, the disease status at transplant and the clinical infection history. Forty-eight patients received a single administration of rituximab 375 mg/m^2^ on day −7 before transplant while 3 patients received two separate infusions of rituximab 375 mg/m^2^ due to a very high anti-donor isohemagglutinin titer (375 mg/ m^2^ at day −7 and −1 before transplant).

The control group (80 patients) was made by all consecutive patients with ABO incompatibility transplanted between January 2013 and August 2016 (65 patients) and includes a small series of 15 patients transplanted between September 2016 and July 2019 who did not receive rituximab because of a low anti-donor isohemagglutinins titer before transplant.

Engraftment definitions, transfusion policy, GVHD, infection prophylaxis and intravenous immunoglobulin infusion policy are available in the Supplementary Material.

### Statistical analysis

Categorical characteristics at allogeneic HSCT were reported with absolute and percentage frequencies and compared, between rituximab or no rituximab treatment, with the Chi-squared test or Fisher’s exact test. Continuous variables were presented as median with range or interquartile range (IQR) and compared using the Mann–Whitney *U* test. Overall survival (OS) and Disease-free survival (DFS) were estimated with the Kaplan–Meier method and compared using the log-rank test with 95% confidence intervals (CIs). Cumulative incidence of relapse (CIR), transplant-related mortality (TRM), and relapse mortality (RM) were estimated using the cumulative incidence function, considering death as a competing event for CIR, relapse as a competing event for TRM and transplant-related death as competing event for RM, using Gray’s test to assess differences between groups. The median time to RBC transfusion independence, neutrophil engraftment, and platelet engraftment was estimated with the cumulative incidence function, considering death as a competing event. To calculate time to transfusion independence patients were also censored at the time of eventual relapse, sepsis, or grade IV acute GVHD. Univariate and multivariable analyses on transfusion independence were performed by fitting Fine-Gray sub-distribution hazard models. The proportional hazard assumption was verified for all estimated models. A hazard ratio with 95% confidence intervals was reported. In this context, the thresholds used to categorize the number of CD34+ infused and the number of total nucleated cells infused were identified through receiver operating characteristic analysis, maximizing Youden’s index. Other outcomes were compared between rituximab or no rituximab treatment with chi-squared test or Mann-Whitney U test, as opportune. *P* values were two-sided, with a 5% significance level. Statistics were performed with R software, version 3.5.0.

## Results

### Study population

The main characteristics of the study population are listed in Table 1. The median follow-up was 16 months for the rituximab group and 25 months for the control group. The AB0-match combinations between patients and donors are listed in Supplementary Table 1.

**Table 1:** Patients’ characteristics.

Characteristics	Rituximab, *N* = 51	No Rituximab, *N* = 80	*P*
Age, median (range)	52 (18–70)	52 (20–69)	0.8354
Sex, *n* (%)			0.1927
F	25 (49)	30 (37.5)	
M	26 (51)	50 (62.5)	
Disease, *n* (%)			0.8310
AA	2 (3.9)	2 (2.5)	
ALL	5 (9.8)	11 (13.8)	
AML	25 (49)	38 (47.5)	
CML	3 (5.9)	2 (2.5)	
MDS	4 (7.8)	10 (12.5)	
MM	1 (2)	3 (3.8)	
Myelofibrosis	6 (11.8)	5 (6.2)	
CLL / NHL	5 (9.8)	9 (11.2)	
Disease status, *n* (%)			0.9592
1°RC	25 (49)	40 (50)	
2°RC	6 (11.8)	6 (7.5)	
>2°RC	1 (2)	3 (3.8)	
active disease	14 (27.5)	21 (26.2)	
RP / stable disease / clinical improvement	3 (5.9)	6 (7.5)	
First CP / never treated	2 (3.9)	4 (5)	
*N* previous lines, median (range)	1 (0–4)	1 (0–6)	0.6275
0	4 (7.8)	7 (8.8)	
1	30 (58.8)	42 (52.5)	
2	11 (21.6)	21 (26.2)	
>2	6 (11.7)	10 (12.6)	
IgG (mg/dl), median (range)	812 (168–1640)	954.5 (323–1370)	0.1624
Serum Gamma globulins (g/dl), median (range)	0.8 (0.2–1.5)	0.9 (0.3–1.4)	0.0612
Conditioning, *n* (%)			0.5904
Immunosuppressive	1 (2)	3 (3.8)	
Myeloablative	27 (52.9)	37 (46.2)	
Reduced intensity	22 (43.1)	40 (50)	
Donor, *n* (%)			0.9915
Cord blood	1 (2)	3 (3.8)	
Haploidentical	2 (3.9)	4 (5)	
Ident. sibling	5 (9.8)	8 (10)	
Matched unrelated	26 (51)	41 (51.2)	
Mismatched unrelated	17 (33.3)	24 (30)	
Source, *n* (%)			0.4734
Bone marrow	2 (3.9)	8 (10)	
Cord blood	1 (2)	3 (3.8)	
Peripheral blood	48 (94.1)	69 (86.3)	
CMV serological status patient/donor			0.5110
Positive/Positive	24 (47.1)	36 (45)	
Positive/negative	21 (41.2)	32 (40)	
Negative/positive	3 (5.9)	2 (2.5)	
Negative/Negative	3 (5.9)	10 (12.5)	
ATG, *n* (%)	41 (80.4)	71 (88.7)	0.1853
N CD34+ infused x 10^6/kg, median (range)	4.7 (0.1-6.8)	4.6 (0.1-8.1)	0.8212
Total nucleated cells infused x 10^6/kg, median (range)	6.6 (0.3-14.6)	6.5 (0.3-28.4)	0.8247
Anti-donor isohemagglutinins titer	Rituximab, *N* = 48	No Rituximab, *N* = 59	P
Titer, median (IQR)	1:128 (1:32-1:512)	1:64 (1:16-1:256)	0.1920
<=1:128	29 (60.4)	39 (66.1)	
>1:128	19 (39.6)	20 (33.9)	

### Red blood cell transfusions and engraftment

**Fig. 1:**
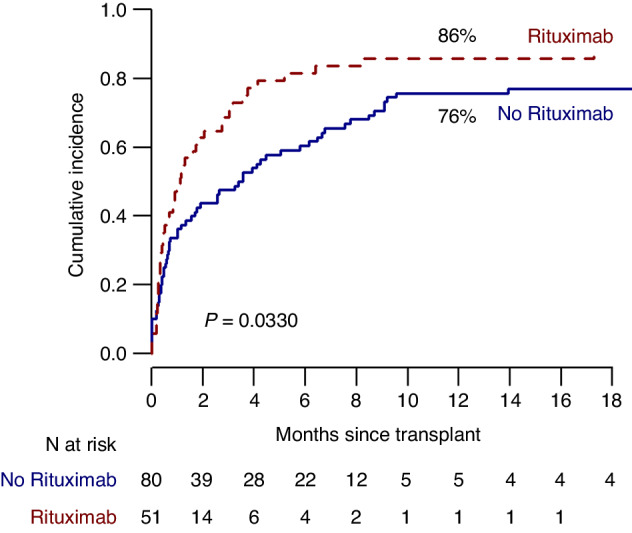
Time to red blood cell transfusion independence. Time to red blood cell transfusion independence after transplant in patients treated with (dotted line) or without (continuous line) rituximab.

**Fig. 2:**
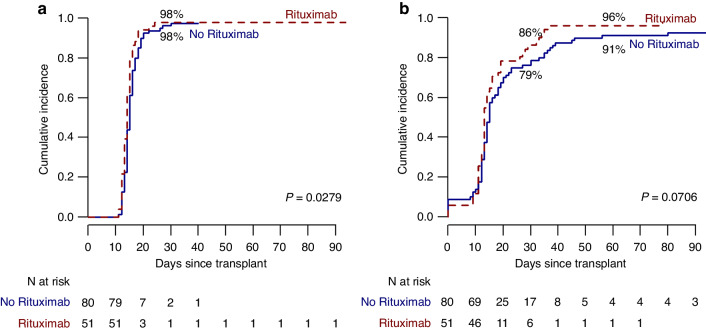
Neutrophil and platelet engraftment. Time to neutrophil (**a**) and platelet (**b**) engraftment in patients treated with (dotted line) or without (continuous line) rituximab.

The median number of RBC units transfused after transplant was 6 (range 0–145) and 8 (range 0–150) respectively in the rituximab and the control group (*p* = 0.22). RBC transfusion independence at 6 months was reached respectively by 41 (80%) and 48 (60%) patients in the rituximab and the control group (*p* = 0.03) (Fig. 1). In addition, the median time to transfusion independence was significantly reduced for patients in the rituximab group (1 month, 95% CI, 0.5–2), compared to the control group (3.2 months, 95% CI 1.5–3.2, *p* = 0.02). The median time to neutrophil engraftment was not clinically different among the two groups (15 days (95% CI, 14–16) and 14 days (95% CI, 13–15) respectively, *p* = 0.03) (Fig. 2a). The median time to platelet engraftment was 15 days (95%, CI 14–18) and 13 days (95% CI, 13–16) respectively for the rituximab and the control group (Fig. 2b, *p* = 0.07).

PRCA was observed in 1 (2%) patient in the rituximab group and 5 (6%) patients in the control group (*p* = 0.49). None of the patients experienced immune hemolytic anemia after transplant. Primary graft failure was observed uniquely in one patient in the control group. A secondary graft failure was documented in 2 patients in the rituximab group and 2 patients in the control group. Two patients in the rituximab group and 3 patients in the control group experienced poor graft function.

### Post-transplant immune reconstitution and infections

**Fig. 3:**
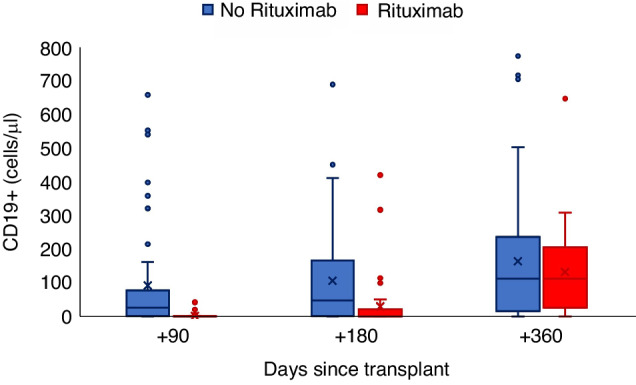
Post-transplant B cell reconstitution. The number of CD19 + B cells/mcl determined at +90 days, +180 days and +360 days after transplant.

At day 90 and 180 after transplant, the median number of peripheral blood CD19 + B cells was significantly inferior for patients in the rituximab group (0/mcl, range 0–42 at day+90 and 1/mcl, range 0–420 at day +180) compared to the control group (27/mcl, range 0–1369 at day +90 and 47/mcl, range 0–689 at day +180). This difference was no longer evident at day +360 (112/mcl, range 0–647 in the rituximab group and 112/mcl, range 0–774 in the control group*, p* = 0.89) (Fig. 3). The number of peripheral blood CD4+ cells was similar in the two groups at any time point (Table 2). The median value of serum gamma globulin at day +90 and +180 was significantly lower in the rituximab group (0.6 g/dl *versus* 0.8 g/dl at day +90, *p* = 0.0002 and 0.4 g/dl *versus* 0.7 g/dl at day +180, *p* < 0.0001), while at day +360 the gamma globulin value was similar in the two groups (0.5 g/dl in the rituximab group and 0.7 g/dl in the control group, Table 2).

**Table 2:** Post-transplant peripheral blood CD19^+^ cells, CD4^+^ cells and gamma globulin recovery.

	Rituximab, *N* = 51	No Rituximab, *N* = 80	*P*
Day + 90			
Total lymphocytes	645 (219–4169)	862 (77–4269)	0.4563
CD19/mcl	0 (0–42)	27 (0–1369)	<0.0001
CD19%	0 (0–9.6)	2.5 (0–107.6)	<0.0001
CD4/mcl	110 (4–971)	119 (3–1011)	0.6247
CD4%	16 (1.3–60.3)	12.5 (2.1–47.1)	0.0958
Gamma_globulin (g/dl)	0.6 (0.3–1.4)	0.8 (0.1–2.1)	0.0002
Day + 180			
Total lymphocytes	1258 (157–3488)	1130 (91.3–6060)	0.5237
CD19/mcl	1 (0–420)	47 (0–689)	0.0001
CD19%	0.1 (0–23.2)	4.1 (0–65.9)	<.0001
CD4/mcl	172 (33–696)	145 (5–3486)	0.3279
CD4%	16.1 (5.6–55)	13.7 (3.2–103.1)	0.5072
Gamma_globulin (g/dl)	0.4 (0.1–1.1)	0.7 (0.1–2)	<.0001
Day + 360			
Total lymphocytes	1838 (320–4027)	1557 (201–8528)	0.2293
CD19/mcl	112 (0–647)	112 (0–774)	0.8929
CD19%	5.9 (0–21.1)	8 (0–46.7)	0.6765
CD4/mcl	315.5 (87–885)	215 (35–2046)	0.0787
CD4%	18.2 (6.8–41.4)	16.6 (5.9–57.5)	0.2075
Gamma_globulin (g/dl)	0.5 (0.1–1.6)	0.7 (0.1–5.3)	0.0809

Intravenous immunoglobulin (IVIG) infusion after transplant was necessary for 32 (63%) patients in the rituximab group and 39 (49%) patients in the control group (*p* = 0.16). The median number of post-transplant IVIG infusions received by patients was 1.5 (1–12) and 2 (1–22) respectively in the rituximab and the control group (*p* = 0.25).

Post-transplant CMV reactivation was detected in 27 (53%) patients in the rituximab group and 15 (19%) patients in the control group (*p* < *0.001*); however, none of the patients in the rituximab group developed CMV disease, while only one patient in the control group developed CMV disease.

Epstein-Barr virus reactivation was detected respectively in 1 (2%) and 7 (9%) of patients in the rituximab and the control group (*p* = *0.15*). None of the patients in both groups developed post-transplant lymphoproliferative disorder (PTLD).

Episodes of infections of any grade (including bacterial, fungal, or viral infections) occurring within the first 180 days after transplant were recorded in 22 patients (43%) in the rituximab group and 36 patients (45%) in the control group.

### Transplant-related complications

Grade II-IV acute GVHD developed in 15 (29.4%) patients in the rituximab group and 23 (28.8%) patients in the control group (*p* = 0.93), while chronic GVHD was observed in 18 (35%) and 29 (36%) patients respectively in the rituximab and the control group (*p* = 0.91). Moderate to severe chronic GVHD was recorded in 12 (24%) and 19 (24%) patients respectively in the rituximab and the control group. Deaths and causes of death are listed in Supplementary Table 2.

### Transplant outcomes

**Fig. 4:**
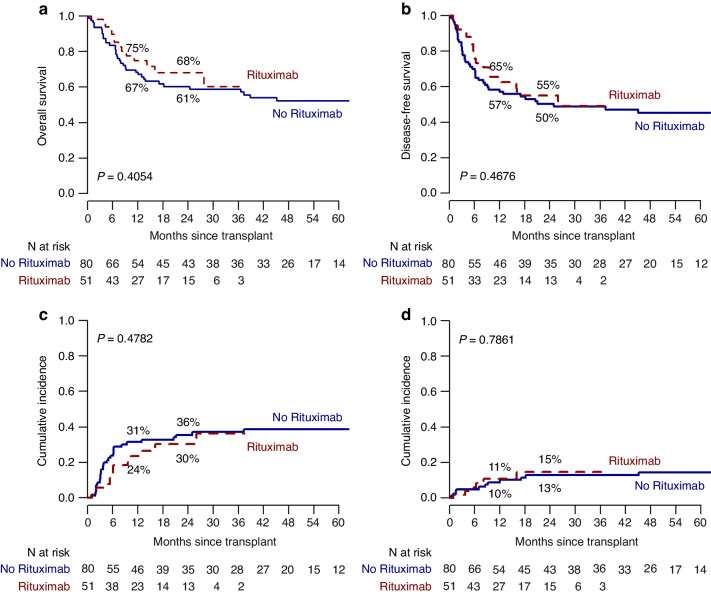
Clinical outcomes according to rituximab added to the conditioning regimen. Overall survival (**a**), disease-free survival (**b**), cumulative incidence of relapse (**c**), and transplant-related mortality (**d**). No rituximab (continuous line), rituximab (dotted line).

There was no difference in OS for patients treated with or without rituximab (respectively, 68 and 61%), (*p* = 0.41) (Fig. 4a). The estimated 2-year Disease Free Survival (DFS) was 55% in the rituximab group and 50% in the control group (*p* = 0.47) (Fig. 4b). The estimated 2-year Relapse Incidence was 30% in the rituximab group and 36% in the control group (*p* = 0.48) (Fig. 4c). Finally, the estimated 2-year Transplant Related Mortality was 15% in the rituximab group and 13% in the control group (*p* = 0.79) (Fig. 4d).

### Factors influencing transfusion independence

On univariable analysis (Table 3), factors positively associated with the reaching of transfusion independence were the use of rituximab (RR 1.5, 95% CI 1.01–2.23 *p* = *0.045*) and having received at transplant a total number of CD34^+^ cells > 3.7 × 10 ^ 6/kg (RR 1.58, 95% CI 1.02–2.45, *p* = 0.039), while a disease status at transplant other than complete remission was a risk factor for delayed transfusion independence (RR 0.54, 95% CI 0.37-0.81, *p* = 0.002 for active disease; RR 0.27, 95% CI 0.09–0.77, *p* = 0.015 for chronic phase). Univariable analysis of the impact of anti-donor isohemagglutinins titer demonstrated that patients with a pre-transplant higher titer (>1:128) were at higher risk for delayed transfusion independence (HR 0.64, 95% CI 0.43–095, *p* = 0.029). We also separately analyzed the impact of anti-donor isohemagglutinins titer in the two study groups and this association was confirmed only in the rituximab group (HR 0.45, 95% CI 0.24–0.86, *p* = 0.016), but not in the control group (HR 0.78, 95% CI 0.46–1.31, *p* = 0.35). By multivariable analysis, rituximab use, disease status at transplant, the total number of CD34+ cells infused at transplant and anti-donor isohemagglutinins titer maintained their prognostic significance on transfusion independence (Table 3).

**Table 3:** Univariable and multivariable analysis of factors influencing time to reach transfusion-independence.

Factors	Univariate		Multivariable	
	HR (95%CI)	*P*	HR (95%CI)	*P*
Rituximab				
No	1		1	
Yes	1.5 (1.01–2.23)	0.045	1.88 (1.17–3.03)	0.009
Age				
<55	1			
>=55	0.84 (0.57–1.23)	0.36		
Sex				
F	1			
M	1.24 (0.84–1.83)	0.28		
Disease				
AML + MDS + CML	1		1	
ALL + CLL + NHL + MM	1.84 (1.18–2.88)	0.0075	3.06 (1.85–5.06)	<0.0001
Myelofibrosis+AA	0.62 (0.35–1.09)	0.0970	0.56 (0.23–1.37)	0.20
Disease status				
RC	1		1	
Active disease	0.54 (0.37–0.81)	0.0024	0.49 (0.30–0.79)	0.003
Chronic phase	0.27 (0.09–0.77)	0.015	0.28 (0.09–0.90)	0.03
Conditioning				
Myeloablative	1			
Immunosuppressive	0.37 (0.11–1.27)	0.11		
Reduced intensity	0.75 (0.52–1.1)	0.14		
Donor				
Ident. sibling	1		1	
Cord blood	2.22 (1.3–3.78)	0.0033	4.99 (1.93–12.88)	0.0009
Haploidentical	0.91 (0.29–2.86)	0.87	2.79 (0.81–9.64)	0.11
Matched unrelated	1.24 (0.73–2.12)	0.42	1.74 (0.81–3.74)	0.16
Mismatched unrelated	1.68 (0.97–2.93)	0.066	1.17 (0.49-2.79)	0.72
ATG				
No	1			
Yes	1.28 (0.72–2.27)	0.41		
N CD34+ infused				
<3.7	1		1	
>3.7	1.58 (1.02–2.45)	0.039	1.88 (1.01–3.48)	0.05
Total nucleated cells infused				
<4	1			
>4	1.53 (0.86–2.73)	0.15		
Anti-donor isohemagglutinins titer				
<=1:128	1		1	
>1:128	0.64 (0.43–0.95)	0.029	0.61 (0.37–0.99)	0.05

## Discussion

In this retrospective, single-center study we originally tested the hypothesis that pre-transplant administration of rituximab in patients with major or bidirectional ABO incompatibility improves RBC engraftment. Adding rituximab to conditioning resulted in a statistically significant reduction of the time to achieve transfusion independence after transplant compared to a historical cohort of patients who did not receive any preventive strategy.

Rituximab is currently used in ABO-incompatible solid organ transplantation as part of desensitization methods [19, 20]. In the setting of hematopoietic stem cell transplantation, current guidelines for the prevention of delayed RBC engraftment and PRCA include the following measures: to reduce graft RBC to ensure an RBC volume <20 mL, pre-transplant plasma exchange or immunoabsorption [15] The use of plasma exchange alone for the reduction of isohemagglutinins seems to have only a temporarily limited effect since the rebound of anti-donor isohemagglutinins titer is described [15]. Pre-transplant immunoadsorption may be performed with an infusion of donor-type fresh frozen plasma positive in A or B antigens to neutralize respective isohemagglutinins [21, 22] or with donor-type RBC transfusion [23, 24]. Although effective, in vivo immunoabsorption requires high expertise in transfusion medicine, and the transfusion reactions reported after this procedure may limit the feasibility of this method.

In our center, plasma exchange or in vivo immunoadsorption do not represent standard procedures in ABO major incompatibility transplants. Only 3 over 55 patients were treated with plasma exchange in the rituximab group, and this prevents us from speculating on a comparison between rituximab and plasma exchange in reducing the time to RBC engraftment.

Another option to treat delayed RBC engraftment after allogeneic stem cell transplantation is Daratumumab, a human monoclonal antibody targeting CD38. In a previous study [25], one patient allografted for myelodysplastic syndrome who developed PRCA and resulted refractory to many treatments was successfully treated with daratumumab. Although intriguing, this result was limited to a single patient, and data about the long-term effects of post-transplant daratumumab are lacking. Furthermore, we tried to explore a prophylactic strategy to prevent the development of delayed RBC engraftment rather than treating the complication itself.

Previous studies reported that, in the context of an ABO incompatible transplant, the anti-donor isohemagglutinin titer was associated with post-transplant transfusion need [17, 26]. Our study confirms this observation since there was a positive correlation between a higher titer (>1:128) and delayed RBC engraftment by univariable and multivariable analysis. We also analyzed this correlation separately in the two groups, and we observed this correlation only in the rituximab group but not in the control group. We hypothesize that the rather lower median anti-donor isohemagglutinin titer of the control group could prevent the finding of a correlation between the titer and the transfusion need. On the other hand, the positive correlation between the anti-donor titer and a late RBC engraftment in the rituximab group suggests that pre-transplant infusion of rituximab alone may be insufficient to prevent a delayed RBC engraftment in patients with an extremely high anti-donor isohemagglutinin titer, where rituximab infusion could be usefully combined with other preventive strategies.

We did not find a significant difference in the incidence of immunological complications such as PRCA or post-transplant immune hemolytic anemia; this observation may be due to the limited number of patients enrolled in this study and requires further studies.

This study has several limitations that deserve to be analyzed, in particular the retrospective and monocentric nature of the study, and the heterogeneity of the patient’s population. Moreover, the decision to administer rituximab in patients with major or bidirectional ABO incompatibility was started in 2016 and since then 15 out of 66 patients did not receive rituximab because of a low anti-donor isohemagglutinins titer. We also acknowledge that the control group was not made up of consecutive patients. Despite this negative selection bias, the rituximab group seemed to have a better outcome concerning time to RBC engraftment compared with the control group, so we may speculate that a comparison between more homogeneous groups should at least confirm the results of this study. A matched analysis for anti-donor isohemagglutinins titer would resolve this issue but it was not statistically feasible due to the small number of patients enrolled.

Despite the better time to RBC engraftment, we could not demonstrate a significant reduction in the median number of RBC units transfused after transplant for patients treated with rituximab. We are tempted to speculate that this may be related to the high heterogeneity of the patient populations and the broad variability of the number of RBC units required by the two groups.

The use of rituximab in the peri-transplant setting can potentially increase the infection risk. In our study, patients who received rituximab experienced a significant impairment in the post-transplant CD19^+^ B cells reconstitution up to 180 days after transplant, which completely resolved at day +360 after transplant and the same applied to gamma globulin level. Nevertheless, this result did not translate into a greater necessity to infuse IVIG in the rituximab group, and the gamma globulin level was similar in the two groups at day +360 after transplant.

The B cell reconstitution impairment in the rituximab group was associated with an increased rate of CMV reactivations. With the use of pre-emptive therapy, none of the patients in the rituximab group experienced CMV disease. The problem of CMV reactivation could probably be mitigated by the introduction of prophylaxis with Letermovir, which was not available in the years investigated in this study. Moreover, it must be considered that rituximab and Letermovir prophylaxis significantly increase the economic burden of the transplant procedure.

Previous reports suggested that rituximab administration before transplant may have a role in reducing the risk of both acute [27, 28] and chronic GVHD [29, 30]. In our study, we did not find a significant difference in the incidence of acute or chronic GVHD in the two study groups. This could be explained by the broad use of anti-thymocyte globulin for the prophylaxis of GVHD in the two study groups.

In conclusion, the result of this study suggests that rituximab added to conditioning regimens is feasible, safe, and can improve time to RBC engraftment after a major ABO-incompatible transplant. Additional studies are needed to understand the role of rituximab on infection risk and long-term effects on engraftment and transplant outcome.

### Supplementary information

Supplementary material

## Data Availability

The data that support the findings of this study are available on request from the corresponding author. The data are not publicly available due to privacy or ethical restrictions.
