# Supplementary material for: Rituximab added to conditioning regimen significantly improves erythroid engraftment in major incompatible ABO-group hematopoietic stem cell transplantation
Source: Bone Marrow Transplant. 2024 Feb 24;59(6):751–8. doi: 10.1038/s41409-024-02247-w (PMC11161407; doi:10.1038/s41409-024-02247-w)
Supplement: Supplementary file 1 — Supplementary material [file 41409_2024_2247_MOESM1_ESM.docx]

**SUPPLEMENTARY**

**GVHD prophylaxis and definition**

GVHD prophylaxis included anti-thymocyte globulin (ATG), Ciclosporin-A (CSA), and Methotrexate for transplant from a matched unrelated donor and one antigen mismatched related donor. For the matched related donors, GVHD prophylaxis was performed with CSA and Methotrexate when the stem cell source was bone marrow, and ATG was added when the stem cell source was peripheral blood. In the case of a related haploidentical donor, GVHD prophylaxis included post-transplant Cyclophosphamide (PTCy) at 50 mg/kg/day on days 3 and 4 after allo-HCT, CSA from day +5, and mofetil mycophenolate from day +5 until day +35 after transplant.

The post-transplant development of GVHD was collected from patients’ clinical records. GVHD was defined as acute or chronic according to Filipovich's criteria(1). Staging and grading of acute GVHD was performed according to the MAGIC consortium criteria(2), while chronic GVHD was graded according to the 2014 NIH Consensus criteria(3).

**Engraftment Definitions and Transfusion Policy**

Neutrophil engraftment was defined as the first of 3 consecutive days with an absolute neutrophil count higher than 0.5 × 10^9^/L. Platelet engraftment was defined as the first of 3 consecutive days with a sustained platelet count higher than >20 × 10^9^/L platelets. Patients were defined as transfusion-independent when they did not receive any transfusion for at least three months(4).

Primary graft failure was defined as ANC <0.5 × 10^9^/L by day +28 after transplant, hemoglobin <80 g/L, and platelets <20 × 10^9^/L, with confirmation of donor cell origin required for RIC regimen transplant. Secondary graft failure was defined as ANC <0.5 × 10^9^/L after initial engraftment not related to relapse, infection, or drug toxicity. For RIC transplant, requires the loss of donor hematopoiesis to < 5%(5).

Poor graft function was defined as two or three cytopenias lasting for more than 2 weeks, after day +28 and in the presence of donor chimerism >95%.

Major ABO incompatibility was defined as recipient 0 / donor A, B, or AB; recipient A/donor AB; recipient B/ donor AB; bidirectional incompatibility was defined as recipient A/donor B; recipient B / donor A.

The hemoglobin threshold for red blood cell transfusion was 80 g/L. Patients with cardiac comorbidities or symptomatic dyspnea were transfused to keep hemoglobin level > 90 g/L. All blood components were white blood cell-depleted and irradiated. The blood group choice for transfusions was made according to the published standard recommendation for transfusions in ABO-incompatible HSCT(5).

Pure red cell aplasia (PRCA) was defined as anemia, reticulocytopenia, and red blood cell transfusion dependence together with bone marrow aspirate or biopsy showing isolated depletion or nearly depletion of erythroid precursors in otherwise normal marrow(6).

**Infection prophylaxis**

Monitoring of cytomegalovirus (CMV) infection was performed by detection of CMV-DNA on peripheral blood two times weekly until day +100 after transplant, and then on a patient-based schedule. CMV reactivation was defined as detection of CMV-DNA ≥1000 copies/ml. CMV reactivation was treated with pre-emptive therapy according to current guidelines(7). None of the patients received Letermovir as CMV-reactivation prophylaxis since it was not available yet during the observation time of the study.

Patients with hypogammaglobulinemia (serum IgG level <0.4 g/dL) and recurrent infections received intravenous immunoglobulin infusions (IVIg) at a dosage of 400 mg/kg body weight every 4-6 weeks until resolution of hypogammaglobulinemia.

**REFERENCES**

1. Filipovich AH, Weisdorf D, Pavletic S, Socie G, Wingard JR, Lee SJ, et al. National Institutes of Health Consensus Development Project on criteria for clinical trials in chronic graft-versus-host disease: I. diagnosis and staging working group report. Vol. 11, Biology of Blood and Marrow Transplantation. 2005. p. 945–56.

2. Harris AC, Young R, Devine S, Hogan WJ, Ayuk F, Bunworasate U, et al. International, Multicenter Standardization of Acute Graft-versus-Host Disease Clinical Data Collection: A Report from the Mount Sinai Acute GVHD International Consortium. Biology of Blood and Marrow Transplantation. 2016 Jan 1;22(1):4–10.

3. Jagasia MH, Greinix HT, Arora M, Williams KM, Wolff D, Cowen EW, et al. National Institutes of Health Consensus Development Project on Criteria for Clinical Trials in Chronic Graft-versus-Host Disease: I. The 2014 Diagnosis and Staging Working Group Report. Biology of Blood and Marrow Transplantation. 2015 Mar 1;21(3):389-401.e1.

4. Gale RP, Barosi G, Barbui T, Cervantes F, Dohner K, Dupriez B, et al. What are RBC-transfusion-dependence and -independence? Leuk Res [Internet]. 2011 [cited 2023 Feb 1];35(1):8–11. Available from: https://pubmed.ncbi.nlm.nih.gov/20692036/

5. EBMT Handbook | EBMT [Internet]. [cited 2023 Feb 1]. Available from: https://www.ebmt.org/education/ebmt-handbook

6. Aung FM, Lichtiger B, Bassett R, Liu P, Alousi A, Bashier Q, et al. Incidence and natural history of pure red cell aplasia in major ABO-mismatched haematopoietic cell transplantation. Br J Haematol. 2013 Mar;160(6):798–805.

7. Ljungman P, de la Camara R, Robin C, Crocchiolo R, Einsele H, Hill JA, et al. Guidelines for the management of cytomegalovirus infection in patients with haematological malignancies and after stem cell transplantation from the 2017 European Conference on Infections in Leukaemia (ECIL 7). Lancet Infect Dis. 2019 Aug 1;19(8):e260–72.

**Supplementary Table 1** *AB0-blood group combinations between patient and donor*

| **Patient** | **Donor** | **Rituximab-group**  **n=51 (%)** | **Control –group**  **n=80 (%)** |
| --- | --- | --- | --- |
| **0** | **A** | 29 (56.9) | 41 (51.3) |
| **0** | **B** | 11 (21.6) | 11 (13.8) |
| **0** | **AB** | 2 (3.9) | 1 (1.2) |
| **A** | **AB** | 3 (5.9) | 3 (3.7) |
| **A** | **B** | 4 (7.8) | 6 (7.5) |
| **B** | **A** | 2 (3.9) | 17 (21.3) |
| **B** | **AB** | 0 | 1 (1.2) |

**Supplementary Table 2.** Deaths and causes of death.

|  | **Rituximab (n=51) n, (%)** | **No Rituximab (n=80) n, (%)** |
| --- | --- | --- |
| **Deaths** | 14 (27%) | 36 (45%) |
| Disease relapse | 8 | 27 |
| Infection | 5 | 4 |
| GVHD | 1 | 2 |
| Encephalopathy | 0 | 2 |
| Hemorrhage | 0 | 1 |
